# Bustling argon: biological effect

**DOI:** 10.1186/2045-9912-3-22

**Published:** 2013-10-03

**Authors:** Zhouheng Ye, Rongjia Zhang, Xuejun Sun

**Affiliations:** 1 Department of Diving Medicine, Second Military Medical University, Shanghai 200433, China

## Abstract

Argon is a noble gas in group 18 of the periodic table. Certificated to exist in air atmosphere merely one century ago, discovery of argon shows interesting stories of researching and exploring. It was assumed to have no chemical activity. However, argon indeed present its biological effect on mammals. Narcotic effect of argon in diving operation and neur-protective function of argon in cerebral injury demonstrate that argon has crucial effect and be concentrated on is necessary. Furthermore, consider to be harmless to human, argon clinical application in therapy would be another option.

## Introduction

In periodic table, helium (He), neon (Ne), argon (Ar), krypton (Kr), xenon (Xe), and the radioactive radon (Rn) make a group of chemical elements with extraordinary properties. Under standard conditions, these elements are all monatomic gases with very low chemical reactivity in a full valence electron shells. Thus we call these elements the “noble gases” according to their stillness property [1]. But it recently turns out that these noble gases are not completely inactive. Based on their relatively rare characteristic, noble gases gained another alternative name called “rare gases”. In the history of scientific research, it is comfortable to read a lot of romances of discovering rare gases.

Tracing to one century ago, argon was verified for air existence. Argon has the property that its atomic number is 18 and its symbol in periodic table is Ar. Argon is the third dominant component in atmosphere right after nitrogen and oxygen. It is colorless and odorless. Atomic number of argon is 18. At zero degree condition, the density of argon is 1.784 g/L, a slightly higher than density of nitrogen which is 1.251 g/L. At 27 degree condition, the coefficient of thermal conductivity of argon is 0.0178 W/mK while the coefficient of thermal conductivity of nitrogen is 0.0260 W/mK. These are basic physical properties of argon [1]. However, the biological effect of this gas has not been reported before. In last few years, some articles indicate that the noble gas argon may have diverse biological effect and crucial value in clinical application. This review describes the discovery, biological effect and clinical application of argon.

## Discovery of argon

In 1785, a well-known Britain chemist Henry Cavendish who uncovered hydrogen tried to completely remove oxygen and nitrogen from air (People at that time already known that major components of atmosphere are nitrogen and oxygen). He pumped excess oxygen into a certain amount of air. And he turned nitrogen and oxygen into nitric oxide through loss-of-charge method. Then he absorbed nitric oxide with aqueous alkali and removed excess oxygen through reaction of copper and oxygen. It is interesting that there was still a great amount of gas remained even after removed nitrogen and oxygen from the air. Mr. Cavendish suspected that another element exists in the air, but he was unable to identify what it was. He reported this experimental result but got few attentions from other chemists by then. Even Henry himself didn’t improve this hypothesis any more. In fact, numbers of chemical elements of another Group of periodic table are hiding in this mysterious “remained gas”. Therefore, Lord Cavendish lost the opportunity of finding new chemical elements.

A century passed after that, British physicist Lord Rayleigh III found that the density of nitrogen was 1.2572 g/L while the measurement of quantity of nitrogen through purified nitride was 1.2507 g/L when he analyzed different density of gas from air. Though only a small difference on the third decimal, it was an unacceptable range of experimental error at that time. Rayleigh couldn’t explain it in a reasonable way and published this experimental result to public for a wiser answer. Later, Sir William Ramsay participated in that study. After their repeat and accurate survey, a new element was obtained from the “remained gas”. It constitutes 1% air volume, heavier than nitrogen and has new spectroscopy result. At 1894, Lord Rayleigh and Sir William Ramsay announced this discovery and settle a fresh name for this new gas---“argon”, from the Greek “argos”, meaning lazy. In Chinese, it is “氩”, pronounced as “ya” [2]. This is the story of “The victory of the third decimal”.

## Narcotic effect of high-pressure argon

Diving medical research in 1930s was the earliest observation on biological effect of argon whose objection was to explore inhale gas application in diving. It is no obvious difference between hyperbaric nitrogen and hyperbaric argon in respiration resistance and psychological effect after bold and participating experiment executed by diving medicine researchers themselves. However, the anesthetic effect of argon makes a huge distinction in comparison of argon and nitrogen and exhibits obvious species differences. For instance, most mice emerged evident anesthetic effect after breathing argon at 15.2 atm but rat appear the same effect after receiving 27.0 atm hyperbaric nitrogen. At normal pressure, naturally, argon exhibits no anesthetic effect.

## Organ protection effect of argon

Neuro-protective function of argon was the most researched in field of organ protection. Protective effect of argon on nerve cells was first described by Russian scientists. They reported that hypoxia cerebral injury caused by breathing hypoxia gas mixture was alleviated by argon inspiration. Soldatov found that argon in different concentration(from 25% to 77%) could dramatically elevate livability of experimental animal in hypoxia circumstance and obtain an decline in brain injury caused by hypoxia [3]. Imperfect mortal capability of intricate operation and mathematical operation can also be improved by breathing gas mixture of certain percentage of argon and oxygen with hypoxia circumstance [4]. Newborn rat inhale 21% oxygen, 5% carbon dioxide and 74% argon gas mixture consequently could notably alleviate cochlea auditory hair cell injury caused by cisplatin and gentamicin [5]. This revealed that argon inspiration could ease deafness caused by cisplatin and gentamicin.

In relatively complicated vitro model, Loetscher found that cultured hippocampus cells treated with argon cell got higher livability than control group after oxygen and glucose deprivation and cerebral trauma 50% concentration of argon turn out to be the most powerful protective concentration for injury through comparison in different degree as 25, 50 and 75 per cent [6]. Breathing Argon 3 hours after brain injury still appear the neuroprotection for alleviating brain damage [6]. This application after injury occur pointed that potential value of clinical application of argon in field of treatment for nerve system. All results above were obtained in vitro circumstances.

In accordance to previous vitro experiments, Ryang reassured neuroprotection effect of argon in vivo model. A filament was inserted into cerebral vessel to induce 1 hour focal cerebral ischemia- 24 h reperfusion model in rat. Animals were breathing of 50% argon and 50% oxygen through mask inspiration in treatment group and the control group was received with 50% oxygen and 50% nitrogen treatment. After rat sacrifice, group with argon treatment shows beneficial effects on the number of nerve cells, cerebral infarction volume and mobility of the animal compared with the control group. This results demonstrate that argon inhalation provide a protective effect for brain ischemia-reperfusion injury [7]. N-methyl-D-aspartate (NMDA) induce nerve injury via glutamic acid NMDA receptor overactivation [8]. David implied that inspiration with 50% argon could obvious alleviate NMDA injury and cerebral injury caused by ischemia-reperfusion injury after NMDA injection into corpus striatum or ischemia-reperfusion injury model [9]. He also found that sub-cortex tissue injury will be severer and functional deficiency is not well ameliorated in those animals treated with argon after post-injury cortex protection [9]. These results indicate that, as same as most neuroprotection measures, argon inspiration within ischemia procedure acquire better effect than treatment with argon after ischemia in cerebral ischemia-reperfusion model.

Cardiac arrest can provoke entire cerebral ischemia, and heart recovery can lead to cerebral ischemia reperfusion injury in this entire cerebral neuron damage caused by cardiac arrest model, Brucken demonstrate that recovery after 7 minutes of heart arrest induces obvious neurological dysfunction. After ischemia-reperfusion injury, treatment with 70% argon for an hour show better function recovery than control group after 7 days rest. Meanwhile, group with argon treatment acquire more cortex and hippocampus cells remain than the control group [10]. In addition, neuroprotection of argon also display on cerebral hypoxia ischemia of newborn baby model. Zhuang et al. consider that breathing of 70% argon, 70% helium or 70% xenon could improve moderate hypoxia-ischemia brain damage of which argon emerge the best improvement on hippocampus cell survival after hypoxia following 2 hours noble gas inspiration. Infarct volume was decreased by treatment with argon or xenon, but not helium [11]. These results indicate that argon or xenon imply more strong neuroprotection effect than helium. This is the earliest article which shown a comparison of different gases (argon, helium and xenon) on organ protection.

Previous articles have demonstrated the definite and unified understanding about organ protection effect of argon, but the specific mechanism of argon on organ protection remains unclear. As inactive in chemical property as it is, argon seems to have no possibility of interacting in chemical reaction with other substances in such mild biological system. For that, there is still no reasonable explanation for the biological effect of argon and other noble gas. Some group of scientists speculated that argon active benzodiazepine site which settle on gamma-aminobutyric acid (GABA) receptor leading to GABA receptor effect [12]. Meantime, evidences in vitro suggest that GABA receptor activation exhibit protective effect for neurological system [13]. Applying method of computer simulation, Seto suggested argon can bind with mortal serum albumin enflurane interact site through van der Waals force [14]. Therefore, David makes the conclusion that argon exhibit neuroprotective effect by same mechanism as well as oxygen [9]. Synergistic effect of both argon and oxygen could be another explanation for neuroprotective effect of argon in neurological dysfunction caused by (N-methyl-D-aspartate) NMDA [9]. Regulating NMDA receptor redox pattern, it decrease nerve cells death caused by glutamic acid [15]. In addition, argon elevates cell survival protein expression to against apoptosis may be another explanation for the neuroprotection of argon [16]. In addition, abundance of results demonstrate that noble gases interact with intracellular signaling molecules such as one member of generally common protein family- MAPKs (mitogen-activated protein kinases), extracellular signal-regulated kinase 1/2(ERK1/2). ERK1/2 introduces different function as gene translation, proliferation and differentiation of cell depending on different types of stimulation [17]. Fahlenkamp hypothesizes that argon activates MAPKs kinase to promote ERK1/2 which locate in nerve cells, microglia and astroglia cells [17].

Argon can also protect kidney and heart either, of which researches are not as plentiful as those on neurological protection [18,19].

## Clinical application of argon

In medical aspect, the most fascinating advantage of noble gases will be its no side effect on human. The most powerful impetus in continuing research on biological effect is its harmless feature. Though related researches are in limited scale and number of experiments is not enough, preliminary evidence shows definitely organ protection effect of noble gases. For now, xenon receives relatively most attention on its biological effect compared to other chemical elements in Group 18. Some of these experiments even are good experimental methods of importance to merit detailed today, facilitating access for some critical molecular mechanisms. From practical point of view, however, small anesthetic effect and lower cost would be the most persuasive clinical applicant potential and value of argon. After all, broader, deeper and finer experiments in vivo, in vitro and various animal models need to be applied before clinical application.

It is so curious that noble gases exhibit stunning effect in the field of biology. On one aspect, chemical inactivity make noble gases absolutely no chance of interacting with others in chemical reaction. On the other side, concrete evidence of biological effect is standing in front of us. Biological effect of noble gases not only expresses complicacy of biological system but also challenge Simple Reductionism theory that the phenomenon of life is equivalent to the physical and chemical processes.

## Competing interests

The authors declare that they have no competing interests.

## Authors’ contributions

ZY carried out the paper research and drafted the manuscript. All authors read and approved the final manuscript.
